# Microsurgical laser resection of brainstem cavernous malformations

**DOI:** 10.3171/2019.7.FocusVid.19163

**Published:** 2019-07-01

**Authors:** Ephraim W. Church, Gary K. Steinberg

**Affiliations:** Department of Neurosurgery and Stanford Stroke Center, Stanford University School of Medicine, Stanford, California

**Keywords:** cavernous malformation, cavernoma, brainstem, microsurgery, laser, video

## Abstract

This operative technique video demonstrates laser microsurgery for brainstem cavernous malformations (CMs). In case 1 we demonstrate CO_2_ laser microsurgery for a symptomatic pontine CM using far lateral craniotomy and olivary zone entry. Case 2 demonstrates the subtemporal approach and removal of a paratrigeminal CM, and case 3 is a dorsal midbrain CM. We illustrate several advantages of laser microsurgery including improved visualization in narrow corridors, precise cutting with reduced thermal damage, and effective sealing of small vessels. Over the past decade at Stanford University School of Medicine, over 120 brainstem CMs have been removed using laser microsurgery with good results.

The video can be found here: https://youtu.be/DwwqWGv_vzo.

## Transcript

In this operative technique video we demonstrate microsurgical laser resection of brainstem cavernous malformations in 3 example cases. We started using the CO_2_ laser for eloquent cavernomas in 2009. Over the past decade at Stanford more than 120 brainstem cavernomas have been treated with laser microsurgery with good results (Pandey et al, 2013).

0:46 Advantages of laser cavernoma removal. In the following videos we demonstrate several advantages of laser over traditional microsurgery techniques. These include improved visualization in narrow corridors and its precision along with reduced thermal damage. Disadvantages include hemostasis for larger vessels and operating around corners (Choudhri et al, 2014).

1:08 Example 1, pontine cavernoma. This 44 year old female with familial cavernomas presented with progressive neurological symptoms. A right far lateral craniotomy with neuronavigation and neurophysiological monitoring was planned. Other approaches include retrosigmoid and telovelar if the cavernoma came to the floor of the 4th ventricle. Working superiorly through the vagoaccessory triangle, hemosiderin stained pia was seen. Motor fibers were found anterior to this and a safe entry zone was identified. This case illustrates how the laser allows for microdissection in narrow corridors. The 2 cm cavernoma was removed through a 2 mm opening deep in the cerebello medullary space.

2:20 Example 2, lateral midbrain cavernoma. This 42 year old female with familial cavernoma syndrome presented with severe headaches. A subtemporal approach with neuronavigation and neuromonitoring was planned. Mannitol aided brain relaxation as the temporal lobe was retracted superiorly. We routinely use neuronavigation linked with the microscope. The 4th cranial nerve was identified and tentorium was divided posterior to the nerve’s entry. As the tentorium was divided back to the petrous ridge, the partially exophytic cavernoma became visible. Fibers of the 5th cranial nerve were identified superior and anterior to the cavernoma. The laser was set to 5W. This case illustrates how the CO_2_ laser allows for very precise microdissection with minimal thermal damage to surrounding tissues. The trigeminal nerve was stimulated after removal of the cavernoma and was unharmed, and the patient had a normal neurological exam.

3:57 Example 3, dorsal midbrain cavernoma. This 71 year old female presented with sudden onset diplopia and was found to have a left 4th cranial nerve palsy. We planned a supracerebellar infratentorial approach with neuromonitoring. Other approaches would include interhemispheric transtentorial if the cavernoma was higher in the midbrain and thalamus. This was a left sided approach. The cavernoma was seen beneath a branch of the superior cerebellar artery. The laser was set to 5W. This case demonstrates how the laser helps with good visualization in a deep corridor along with precise cutting and sealing of small vessels.

5:09 Conclusion. In summary we have demonstrated microsurgical laser resection of brainstem cavernomas and the several advantages of this technique over traditional microsurgical techniques. More than 120 brainstem cavernomas have been treated at Stanford with this technique with good results (Pandey et al, 2013).
